# Investigation and Identification of Fungal Diseases of *Aloe barbadensis* in China

**DOI:** 10.3390/biology14010089

**Published:** 2025-01-17

**Authors:** Guohui Zhang, Qingjia Wan, Xiangyang Li, Jie Deng

**Affiliations:** 1 School of Life and Health Science College, Kaili University, Kaili 556011, China; lixiangyang@fudan.edu.cn (X.L.); 15085212228@163.com (J.D.); 2 Yunnan Wanlv Biological Co., Ltd., Kunming 550025, China; wanqingjia163@163.com

**Keywords:** *Aloe barbadensis*, fungal disease, pathogen, identification

## Abstract

The *Aloe barbadensis* industry plays an important role in the economic development of Yuanjiang county of Yuxi city in Yunnan province, China. In order to reduce the harm of diseases and ensure the quality of products, the occurrence of *A. barbadensis* was investigated. The pathogenic fungi of wild and cultivated species of *A. barbadensis* were isolated by a tissue separation method, and DNA sequencing was carried out by using the sequence analysis of the ribosomal rDNA-ITS region, and the pathogenic fungi were classified and identified by finally combining morphological observations. The results showed that the main fungal diseases were root rot and leaf rot disease caused by *Fusarium oxysporum*, leaf spot disease caused by *Curvularia lunata*, anthracnose caused by *Colletotrichum boninense*, and brown spot disease caused by *Alternaria alternata* on *A. barbadensis*.

## Simple Summary

*Aloe barbadensis* is an important material in the pharmaceutical and cosmetic industries. However, large-scale industrial cultivation is significantly linked to the occurrence of diseases. This study aims to clarify the types of diseases currently occurring in *Aloe barbadensis* and their pathogenic fungi, with a view to providing a scientific basis for disease prevention and control.

## 1. Introduction

*Aloe barbadensi* is a perennial, evergreen, succulent herb of the Aloe genus in the Liliaceae family, originally from the tropical arid regions of Africa, now widely distributed around the world. Its flowers, leaves, and roots can all be used medicinally, possessing antiviral, anti-AIDS, anticancer, antibacterial, immune-boosting, heat-clearing and detoxifying, bowel-relaxing, liver-clearing, and vision-improving effects [1,2,3]. *A. barbadensis*, as a traditional plant used both medicinally and as food rich in active ingredients, has now received authoritative approval from the U.S. Food and Drug Administration and the medical community. The various active ingredients studied include anthraquinone compounds, amino acids and organic acids, antibiotics, and saponins [4], among which anthraquinone compounds contain the functional group phenolic hydroxyl, found in natural antioxidants and which has antibacterial effects. Therefore, aloe anthraquinone compounds and anthraquinone derivatives have a strong inhibitory effect on various fungi and bacteria [5], mainly including aloin, aloe-emodin, aloesin, rhein, etc. In addition, rhein in aloe-emodin also has anti-inflammatory, antibacterial, and bactericidal functions. Medically, aloe also contains flavonoid compounds, which have functions similar to superoxide dismutase, possessing certain antioxidant activity and free radical scavenging functions. Antioxidant active substances, including flavonoids in aloe peel extracts, can be used to maintain appropriate levels of free radicals and scavengers, thereby delaying aging [6]. Current research shows that *A. barbadensis* exhibits excellent effects in antioxidation [7,8], anti-tumor effects [9], anti-inflammatory effects [10,11,12], immune regulation [9,13], and wound healing effects, [9,12,14] among others.

Considering the multifaceted importance of *A. barbadensis*, reducing the occurrence of diseases during its cultivation and production is particularly crucial. However, there are currently few research reports available on this aspect. There are many reports of aloe fungal diseases in various regions, but there is relatively little research on the diseases of *A. barbadensis*, and the pathogens exhibit diversity. Existing studies indicate that, in Sri Lanka, both cultivated and wild *A. vera* are susceptible to leaf spot and tip necrosis. These isolates appeared to share a common ancestor with *Lasiodiplodia hormozganensis* [15]. In February 2022, leaf zonate spot disease affected *Aloe vera* in Yunnan, China, endangering the USD 39 billion industry with 600 ha under cultivation. The disease incidence ranged from 10 to 15% in three commercial plantations. Based on its characteristics, the fungus was identified as *Neoscytalidium dimidiatum* [16]. The most research reports are on leaf spot disease. In 2020, the spot disease was first observed on *A. barbadensis*. This is the first report of leaf spot on *A. barbadensis* caused by *A. tenuissima* in Beijing, China [17]. Other reports indicate that the pathogens of leaf spot disease also include *Curvularia spicifera* [18], *Lasiodiplodia theobromae* [19], *Alternaria alternata* [20,21,22], *Alternaria tenuissima* [23], and *Curvularia lunata* [24,25]. Soft rot is a devastating disease in aloe. Previous works have confirmed the identity of its pathogens as *Dickeya zeae* and *Fusarium falciforme* [26]. In 2018 and 2019, a root rot disease emerged in potted *Aloe vera* plants housed in a nursery located in Hunan Province, China. The fungus was tentatively identified as *Fusarium xylarioides* [27]. In June 2014, aloe plants that were one year old and cultivated directly in sandy loam soil in a greenhouse located in Weihai City (Shandong Province) exhibited the presence of up to 5% stunted plants with symptoms characterized by water-soaked lesions on the roots and stem bases. The irrigation method employed was furrow irrigation. The occurrence of stunted plants was initially observed in low-lying areas prone to water accumulation. *Pythium spinosum* was exclusively isolated from the water-soaked roots and stem bases of inoculated plants, and subsequent identification involved morphological characteristics assessment, as well as sequencing analysis targeting the COI gene [28]. Among the diverse fungal diseases was black spot disease, attributed to *Colletotrichum gloeosporioides* [29].

To understand the types of diseases and their pathogenic fungi affecting the *A. barbadensis* production area in Yuanjiang County, Yunnan Province, China, this study conducted a survey of disease types and indoor pathogen isolation and identification in the production area, aiming to provide scientific support for subsequent disease prevention and control efforts.

## 2. Materials and Methods

The instruments used include a sterile workbench, an inoculation needle, scissors, tweezers, a high-pressure steam sterilizer, a constant temperature incubator, a microscope, an induction cooker, a microwave oven, etc. The reagents used include 0.1% mercuric chloride solution, 75% ethanol, sterile water, and industrial alcohol.

The experimental culture medium was PSA medium: peel 200 g of potatoes, slice and boil in water for 20 min, filter, and adjust the filtrate volume to 1000 mL. Add 17 g of sucrose and 17 g of agar, heat to dissolve, and sterilize at high temperature.

### 2.1. Disease Specimen Collection and Symptom Observation

In June 2023, a field survey was conducted at the Yuanwa Road planting base in Yuanjiang Hani, Yi, and Dai Autonomous County, Yuxi City, Yunnan Province. Several rows of plants were randomly selected in the field for collecting diseased plants. The morphology, color, and severity of damage of the diseased leaves (roots) of *A. barbadensis* at the affected parts were observed and described. Photographs of the affected areas were also taken. The occurrence of disease symptoms was recorded, and fresh specimens were collected for pathogen isolation and identification at the Plant Pathology Laboratory of Kaili University.

### 2.2. Isolation, Purification, and Identification of Pathogens

The tissue isolation method was employed to isolate pathogens from the diseased leaves and roots of *A. barbadensis*. Specifically, three pieces of diseased tissue were cut with scissors and first soaked in a 0.1% mercuric chloride solution for 2–3 min, followed by a 2–3 s soak in 75% ethanol. Using forceps, the tissue pieces were arranged in a triangle-shaped pattern on PSA (potato sucrose agar) solid medium. The plates were then incubated inverted at 25 °C in a constant temperature incubator for 5–7 days.

Purification of the resulting colonies was performed twice using an inoculation needle to ensure pure cultures were obtained. From the purified colonies, a central portion of mycelium was placed onto a glass slide. Under a microscope, the morphological characteristics of the pathogen were observed and photographed, and measurements of the mycelia and spore sizes were recorded.

DNA sequencing was conducted using the ribosomal rDNA-ITS (Internal Transcribed Spacer) region sequence analysis method. The ITS sequence results were compared with sequences in the GenBank database using BLAST analysis. Finally, the pathogen was classified and identified by combining molecular data with morphological observations and constructed dendrograms.

### 2.3. Pathogenicity Test

The purified pathogenic fungi were initially cultured on potato sucrose agar (PSA) medium under sterile conditions and incubated at 25 °C for 5–7 days to promote sporulation. After incubation, a spore suspension was prepared by gently washing the fungal spores off the PSA medium surface using sterile distilled water. The spore concentration was adjusted to 10^6^ spores/mL using a hemocytometer to ensure consistency and reproducibility in the inoculation process. This concentration is widely accepted in the literature as it provides reliable results while minimizing the risk of overwhelming the host system. For field inoculation, the spore suspension was applied to *A. barbadensis* leaves and roots using the needle puncture method. In the needle puncture method, small wounds were created on the leaves or roots using a sterile needle, after which 10–50 µL of the spore suspension was directly applied to the wound site. Control plants were treated with sterile distilled water following the same procedure. After inoculation, the plants were monitored daily, and lesion development on the leaves and roots was recorded. Once visible disease symptoms appeared, tissue samples were collected from the infected areas. These diseased tissues were cut with scissors and first soaked in a 0.1% mercuric chloride solution for 2–3 min, followed by a 2–3 s soak in 75% ethanol, and rinsed three times in sterile distilled water to remove surface contaminants. The sterilized tissues were then placed on PSA medium and incubated at 25 °C to allow for fungal growth. The resulting fungal isolates were purified through single-spore isolation and subsequently identified using morphological characteristics and molecular analysis.

## 3. Results

### 3.1. Root Rot and Leaf Rot of Aloe barbadensis

**Figure 1:**
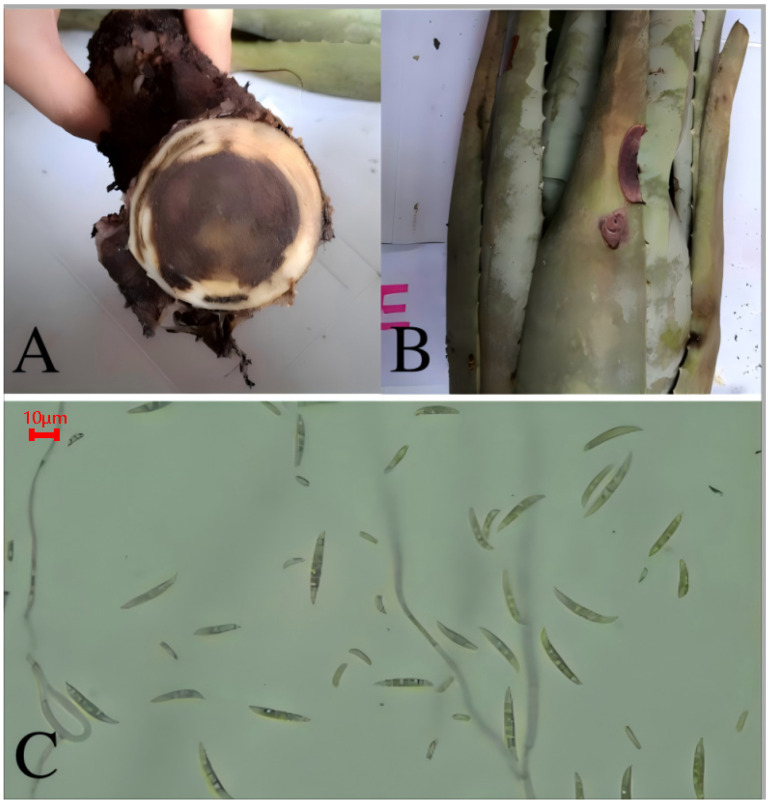
Symptoms of root rot (**A**), leaf rot (**B**), and macroconidia of the pathogen *Fusarium oxysporum* (**C**) from *A. barbadensis* (10 × 40).

The main harm was to the underground roots (and leaves), and occurred in June. When the roots are damaged, the main effect is that the middle part of the roots expands towards the edges, the affected area turns black, and, in severe cases, up to two-thirds of the root area turns black (Figure 1A). The boundary between the diseased and healthy areas is clear. This disease develops rapidly and can cause the roots of *A. barbadensis* to rot and deteriorate, spreading rapidly from the center of the affected area to the surrounding areas in the field. When the leaves are affected, the main point of damage is located at the leaf margin, and appears as semi-circular lesions that spread from the edges to the middle of the leaf. The lesions are grayish-black, with the center being darker and depressed (Figure 1B).

The fungal hyphae are transparent, with septa, and produce spores on lateral conidiophores. The macroconidia are colorless, sickle-shaped, slightly curved, and taper towards both ends, with one–three septa, but mostly with three septa. The spore size is (21.05–28.80) μm × (2.55–4.09) μm (Figure 1C). Based on morphological identification and ITS sequence analysis, the pathogen was identified as *Fusarium oxysporum*, belonging to the fungal phylum, the subphylum Ascomycotina, the order Hypocreales, the family Nectriaceae, and the genus *Fusarium*.

### 3.2. Leaf Spot Disease of Aloe barbadensis

**Figure 2:**
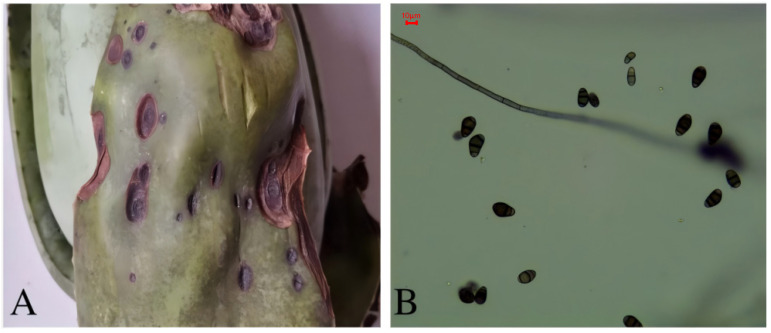
Symptoms of aloe leaf spot disease (**A**) and the pathogen *Curvularia lunata* (**B**) from *A. barbadensis* (right) (10 × 40).

The disease primarily affects the leaves of the plant and occurred in June. The lesions are 0.5–2.5 cm in size. In the early stages of infection, the lesions appear as small, depressed, black, elliptical spots. As the disease progresses, the lesions expand to form larger, depressed, brown, elliptical spots with a black center. In some cases, the lesions may merge to form a single large lesion. The affected areas are depressed and dry, with a hard texture (Figure 2A).

The conidia of the pathogen are solitary, terminal, and often curved to one side. They have three transverse septa, are brown in color, and typically have lighter-colored cells on either side. The conidia measure (16.35–25.98) μm × (8.35–10.19) μm in size (Figure 2B). Based on morphological identification and ITS sequence analysis, the pathogen was identified as *Curvularia lunata*, a species of fungus that causes leaf spot disease in *A. barbadensis*.

### 3.3. Anthracnose Disease of Aloe barbadensis

**Figure 3:**
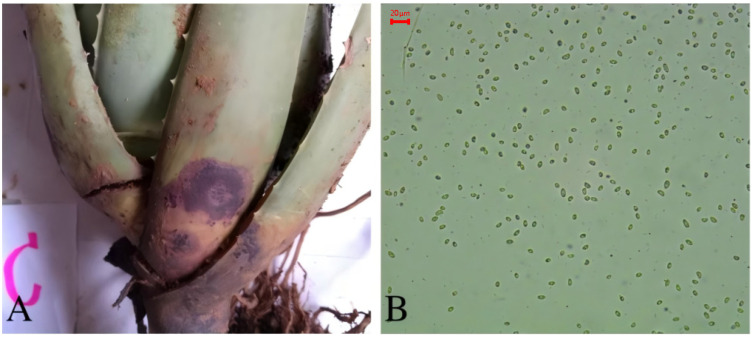
Symptoms of anthracnose (**A**) and the pathogen *Colletotrichum boninense* from *A. barbadensis* (**B**) (10 × 40).

The disease primarily affects the base of the leaves and occurred in June. The initial symptoms appear as brown, water-soaked lesions, which are circular or elliptical in shape. The center of the lesion is light brown, and the affected area rapidly expands and becomes depressed, with the center turning deep brown and the edges slightly lighter in color (Figure 3A). The fungal colonies are cottony, flat, and orange-yellow on the reverse side. The mycelium is sparse, initially white, and later turns dark brown. The conidia are unicellular, colorless, and cylindrical, with most having one end rounded and the other end slightly pointed. The conidia contain two oil droplets and measure (7.61–10.58) μm × (5.06–8.20) μm in size (Figure 3B). Based on ITS sequence analysis and morphological identification, the pathogen was identified as *Colletotrichum boninense*, a species of fungus that belongs to the phylum Fungi, the subphylum Ascomycotina, the order Glomerellales, the family Glomerellaceae, and the genus *Colletotrichum*.

### 3.4. Black Spot Disease of Aloe barbadensis

**Figure 4:**
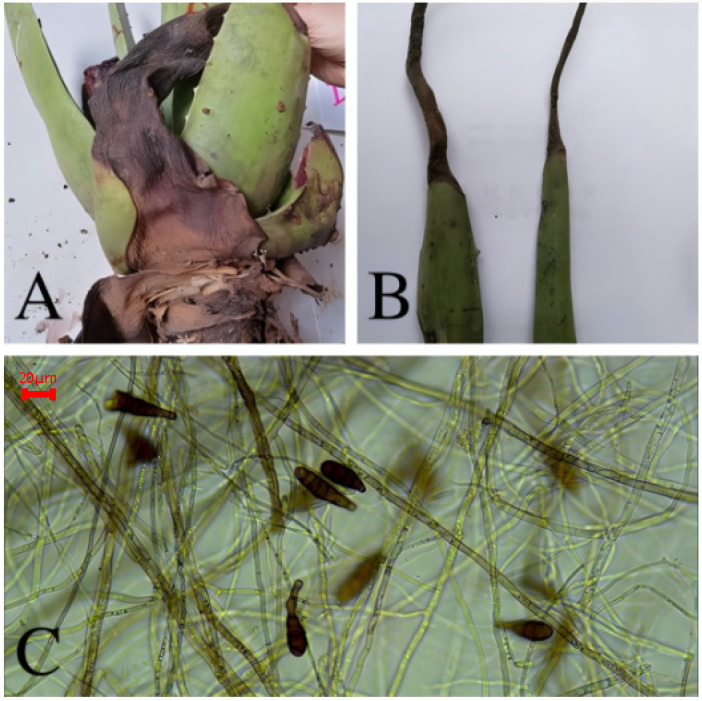
Symptoms of black spot disease (**A**,**B**) and the pathogen *Alternaria tenuissima* (**C**) from *A. barbadensis* (10 × 40).

The disease is more severe in June. The lesions occur on the leaves, initially appearing as water-soaked, dark green spots. In the later stages, the lesions expand along the leaf edges or inward, forming irregular brown spots. In severe cases, the lesions gradually change from brown to black, leading to rot. Under normal climatic conditions, the edges of the brown spots are dark brown, while the center is slightly lighter (Figure 4A). The conidiophores of the pathogen are brown, robust, solitary, and septate. The conidia are either in chains or solitary, oval, inverted pear-shaped, or inverted rod-shaped, measuring (13.50–22.58) μm × (3.62–4.39) μm, and are brown with three–four transverse septa and one–four oblique septa, with constrictions at the septation points (Figure 4B). The ITS region sequence results were compared using BLAST in the GenBank database, and, combined with morphological characteristics, the pathogen was identified as *Alternaria tenuissima*.

**Figure 5:**
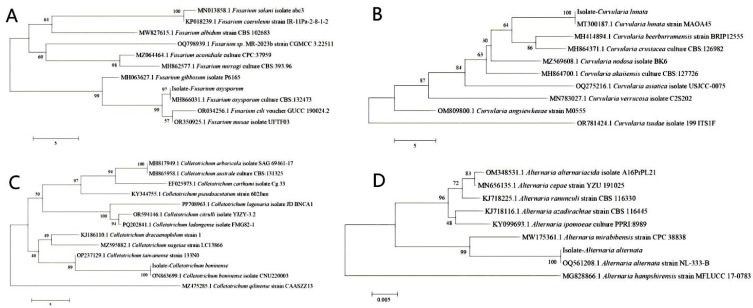
The dendrograms of four pathogens. (**A**) Fusarium oxysporum, (**B**) Curvularia lunata, (**C**) Colletotrichum boninense, and (**D**) Alternaria alternata.

To facilitate understanding and comparison of the four diseases, a summary of the four diseases and their pathogenic fungi is presented in Table 1, and we analyzed the four pathogenic fungi using dendrograms (Figure 5).

### 3.5. Pathogenicity Test

The prepared 10^6^ spores/mL suspension was inoculated into the roots and leaves of *A. barbadensis* using the needle puncture method. After 5–7 days following inoculation with the spore suspension, all four pathogenic fungi caused lesions on the leaves and roots. Pathogenic fungi were then isolated from the diseased leaves (and roots) using tissue isolation methods after 7 days, and then identified microscopically. The results showed that the strains isolated again were the same as the inoculated pathogenic strains. According to Koch’s postulates, this confirms that the inoculated strains are the pathogens causing the diseases in the leaves (and roots) of *A. barbadensis*.

## 4. Discussion

Given the significant medicinal value of *A. barbadensis* and its role as a pillar industry for increasing the income of farmers in suitable growing areas, the lack of knowledge about diseases affecting *A. barbadensis* and the absence of scientific prevention and control techniques have led to increasingly serious disease issues, severely impacting the healthy development of the *A. barbadensis* industry. Currently, there are many reports of aloe fungal diseases in various regions, but there is relatively little research on the diseases of *A. barbadensis*, and the pathogens exhibit diversity [15,16,17,18,19,20,21,22,23,26,27]. Leaf spot disease caused by *Alternaria tenuissima* was observed on *A. barbadensis* for the first time in 2020. This marked the initial report of leaf spot on *A. barbadensis* [17]. Reported diseases of *A. barbadensis* include tip necrosis disease [15], leaf zonate spot disease [16], leaf blight and leaf spot disease [15,17,18,19,20,21,22,23], soft rot disease [26], root and stem rot disease [27,28], and black spot disease [29], among others. There is currently no research on fungal diseases in *A. barbadensis* in Yuanjiang County, Yunnan Province, China. Among them, *C. boninense* was first discovered in *A. barbadensis*. The phenomenon of different pathogens causing the same disease is common in the study of diseases in *A. barbadensis*. Therefore, the identification of the pathogen is very important.

This study focuses on the identification of pathogenic fungi in *A. barbadensis* and holds significant scientific value for the *A. barbadensis* cultivation industry and disease prevention efforts. It professionally identifies the pathogenic fungi responsible for disease occurrence and supplements the list of pathogenic fungi associated with *A. barbadensis*, establishing a solid foundation for future in-depth research.

## Figures and Tables

**Table 1:** The morphological data of four diseases and pathogenic fungi.

Disease Name	Affected Part	Disease Characteristics	Pathogenic Fungi	Morphological Features
Root and leaf rot disease	Roots and leaves	Roots and leaves turn black.	*Fusarium oxysporum*	Conidia are colorless, with one–three septa, (21.05–28.80) μm × (2.55–4.09) μm.
Leaf spot disease	Leaves	Depressed, black, elliptical spots.	*Curvularia lunata*	Brown conidia have three septa, (16.35–25.98) μm × (8.35–10.19) μm.
Anthracnose	Leaves	The center of the lesion is brown.	*Colletotrichum boninense*	Conidia are colorless, with two oil droplets, (7.61–10.58) μm × (5.06–8.20) μm.
Brown spot disease	Leaves	Dark green spots.	*Alternaria alternata*	Brown conidia have three–four transverse septa and one–four oblique septa, (13.50–22.58) μm × (3.62–4.39) μm.

## Data Availability

No new data were created or analyzed in this study. Data sharing is not applicable to this article.
